# Role of Obesity in the Risk of Breast Cancer: Lessons from Anthropometry

**DOI:** 10.1155/2013/906495

**Published:** 2013-02-03

**Authors:** Amina Amadou, Pierre Hainaut, Isabelle Romieu

**Affiliations:** ^1^Nutritional Epidemiology Group, Nutrition and Metabolism Section, International Agency for Research on Cancer, 150 Cours Albert Thomas, 69372 Lyon Cedex 08, France; ^2^International Prevention Research Institute, 95 Cours Lafayette, 69006 Lyon, France

## Abstract

An estimated 1.38 million new cases of breast cancer (BC) are diagnosed each year in women worldwide. Of these, the majority are categorized as invasive ductal cell carcinoma. Subgroups of BC are frequently distinguished into five “intrinsic” subtypes, namely, luminal A, luminal B, normal-like, HER2-positive, and basal-like subtypes. Epidemiological evidence has shown that anthropometric factors are implicated in BC development. Overall consistent positive associations have been observed between high body mass index (BMI) and waist-to-hip ratio (WHR) and the risk of BC among postmenopausal women, while conflicting results persist for premenopausal BC, both for BMI and for other anthropometric parameters as well as across ethnic groups. Furthermore, some evidence suggests that body size, body shape, and weight gain during childhood or adolescence may play a role in the risk of BC. In this paper, we describe the evidence linking anthropometric indices at different ages and BC risk, in order to improve our understanding of the role of body fat distribution in the risk of BC, investigate differences in these associations according to menopausal status and ethnic groups, and discuss the potential biological mechanisms linking body size and BC risk.

## 1. Introduction

Breast cancer (BC) is the most frequently diagnosed cancer in women worldwide, accounting for 23% (1.38 million) of the total new cancer cases in 2008 [1, 2]. The majority of invasive breast neoplasms are categorized as “invasive ductal cell carcinoma, not otherwise specified” (ICD-O 8500/3) [3]. This entity has long been recognized to include tumors with heterogeneous molecular characteristics, characterized by distinct patterns of gene expression [4] and of genomic/genetic alterations [5]. Subgroups of BC are frequently distinguished into luminal A (estrogen/progesterone-positive), luminal B, HER2+, and so-called “triple negative” subtypes [6]. Among these subtypes, HER2+ and basal-like subtypes tend to be more common among premenopausal women, as well as in women of African ancestry. Luminal subtypes are more common in postmenopausal women and among Caucasians [7].

 The incidence rates of BC show a heterogeneous distribution, while Western countries present the highest incidence rates, the lowest incidences are observed in low resource countries. BC ranks as the fifth cause of death from cancer overall (458,000 deaths), but is still the most frequent cause of cancer death in women in both developing (269,000 deaths) and developed regions (189,500 deaths) [1]. Incidence and mortality rates have increased in low-incidence countries, particularly in Latin America (LA) and Africa and especially among younger women. Data from LA show that the highest BC incident rates are among women aged 30–49. In this area, BC is the most common cancer among women, and the leading cause of cancer mortality compounded by problems of access to screening, diagnosis, and treatment [8, 9]. In Mexico for example, BC death has increased 3.6 fold between 1995 and 2005. In Africa, a recent study among Gambian women showed an increase of 6.5% in the incidence rate of BC from 1990 to 2006 [10] compared to 1.5% annually increase worldwide [11]. Asian countries are also recording significant annual increases, for example 2% in Japan and 3–5% in China [11].

The risk factors related to reproductive factors such as delayed childbearing, lower parity, and reduced breastfeeding are becoming more prevalent in countries in economic transition but do not fully explain the increase of BC incidence rates. Other risk factors such as changes in lifestyle, physical activity, and anthropometry as well as ethnicity and genetic susceptibility play a role in the development of BC [12–16]. In particular, overweight and obesity have been clearly associated with an increased overall risk of BC.

Overweight and obesity have become major public health challenges throughout the world in both high and low income countries, with over 1 billion overweight and 315 million obese adults currently estimated worldwide [17, 18]. In the United States (US) for example, obesity is now considered as the second leading cause of preventable death after tobacco consumption. Over two-thirds of the adult population is overweight, with approximately one-third of adults and almost 17% of children and adolescents obese [17, 18]. Most studies on the association between obesity and BC risk have been conducted on Caucasian populations from Europe, Canada, and the US. However, a limited number of studies in other ethnic groups suggest some differences in the prevalence of obesity and fat distribution among women. For example studies in the US show that American women of African or Hispanic origin are more likely to be obese than Caucasian women [19]. Given the disparities in prevalence rates of obesity, it is expected that among women, 69% of Caucasians, 87% of African Americans, and 80% of Hispanics will be overweight in 2015 [19].

Anthropometric indices, height, weight, body mass index (BMI), waist circumference (WC), hip circumference (HC), or waist-to-hip ratio (WHR), are commonly used as tools for assessing overweight/obesity and recently methods for evaluating body shape and body size at different ages have also been used. Several studies and meta-analyses have examined the associations between anthropometric indices and BC among both pre- and postmenopausal women. Overall consistent positive associations have been observed between BMI and WHR and the risk of BC among postmenopausal women [20–29], while conflicting results persist for premenopausal BC, both for BMI and for other anthropometric parameters [29–36]. Possible differences in these associations could be related to differential roles of fat and fat distribution on metabolism and to their contribution to BC development among pre- and postmenopausal women [31, 32]. Waist circumference (WC) and WHR, which correlate with central (abdominal) obesity have been associated with increased BC risk in postmenopausal women [32, 37–39], but not in premenopausal women for whom both null [23, 38, 39] and increased risk have been reported [31, 32, 37, 40]. Furthermore, the relation between premenopausal BC and anthropometry has shown inconsistencies across ethnic groups [41–43]. These inconsistencies may be due to important ethnic variations in body fat distribution. We conducted a systematic review of published studies to evaluate the strength of associations between anthropometric indices at different ages and BC risk in order to improve our understanding of the role of body fat distribution in the risk of BC, to investigate differences in these associations according to menopausal status and ethnic groups, and to highlight the potential biological mechanisms linking body size and BC risk.

## 2. Methods 

To review the epidemiologic literature on the association of overweight, obesity, fat distribution, and BC risk, we conducted a MEDLINE and PUBMED search including all publications using height, weight, BMI, WC, HC, WHR, anthropometric factors, body shape, early life body size, BC, premenopausal, premenopausal BC, case-control, and cohort studies as key words. Our paper follows the preferred reporting items for systematic reviews and meta-analysis (PRISMA) statement guidelines described by Liberati et al. [52]. We then examined the references from the identified articles, previous review and meta-analysis between anthropometric factors and premenopausal and postmenopausal BC. The literature search included all publications from 1997 to 2011 and recent publications up to February 2012.

## 3. Definition, Measurement, and Type of Obesity

Overweight and obesity are defined as abnormal or excessive fat accumulation that may impair health [61]. There are different ways to measure obesity; BMI is the most commonly used and is calculated as weight (kg) divided by height (m²). The World Health Organization (WHO) classifies the degree of adiposity in terms of the BMI as follows: underweight, less than 18.5; normal, 18.5–24.9; overweight, 25.0–29.9; obese, more than 30.0 kg/m² (Table 1(a)). While BMI is usually associated with general obesity, WC and WHR have been used as measures of central or intra-abdominal obesity, defined as waist–hip ratio above 0.90 for males and above 0.85 for females (Table 1(b)). However, there is evidence of important ethnic variations in body fat distribution, especially in Asians as compared to Caucasians. In Asian adults, differences in body build and body composition result in a different relationship between BMI and body fat distribution relative to Caucasians [62–64]. In a study of young Japanese and Australian men, Kagawa and colleagues reported that Japanese men were estimated to have an equivalent amount of body fat as the Australian men at BMI values about 1.5 units lower than those of the Australians (23.5 kg/m² and 28.2 kg/m², resp.) [65]. Similarly, studies among Chinese reported similar relationship between BMI and body fat distribution [43]. These observations suggest that Asian people are more likely to have higher levels of body fat overall and more abdominal fat and lower lean mass than the other ethnic groups for a given BMI [41–43]. Consequently, the level of BMI that the WHO recommends for overweight and obesity in Caucasians may not be applicable for Asian adults. The WHO Western Pacific Regional Office (WPRO) has proposed a definition of obesity in Asian populations with overweight defined as BMI 23.0–24.9 kg/m² and obesity defined as BMI ≥ 25 kg/m². In addition, WHO has suggested that a waist circumference of 90 cm for men and 80 cm for women was used as interim lower values for Asians as the cut-off point to define overweight instead of 94 cm in men and 85 cm for Caucasian women [66].

## 4. In Postmenopausal Women

### 4.1. BMI and Breast Cancer Risk

**Figure 1:**
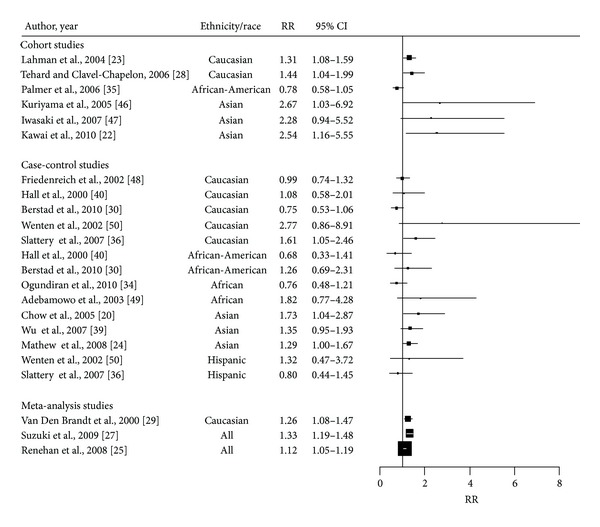
Forest plot of the association between BMI and breast cancer risk in postmenopausal women. The size of each box indicates the relative weight of each study; the horizontal bars show the 95% confidence intervals (CI). RR: relative risk; 95% CI: 95% confidence intervals.

Most available studies and meta-analyses have focused on BMI as a marker of general obesity [22, 24, 25, 27–29, 67] and indicated an overall increase in the risk of postmenopausal BC in overweight or obese women among all ethnic groups (Table 2).  Figure 1 summarizes studies of the association between BMI and breast cancer risk in postmenopausal women. A meta-analysis of some of these studies conducted by Renehan observed an overall 12% increase per 5 kg/m^2^ increase in BMI (RR = 1.12; CI: 1.08–1.16) [25]. However estimates may vary according to geographic regions; the association tended to be stronger in studies from Asia Pacific (RR = 1.31; 95% CI: 1.15–1.48) than studies from North America, Europe, and Australia (RR = 1.15; 95% CI: 1.08–1.23 and 1.09; 95% CI: 1.00–1.14, resp.). Pooled data from seven cohort studies including 337,819 women and 4,385 incident BC cases found a 26% increase in postmenopausal BC risk with BMIs greater or equal to 28 kg/m^2^ [29]. In most case-control and prospective studies, a positive and strong relationship has been observed between BMI and postmenopausal Asian women [20, 22, 67]. In a cohort study of 10,106 women, conducted in Japan, the RR for developing postmenopausal BC was 2.54; 94% CI (1.16–5.55) in women with BMI of 25 kg/m^2^ or above compared to those with less 20.5 kg/m^2^ [22]. In contrast, there is a lack of data among women of African origin, but the notion that increase in BMI is associated with the risk of postmenopausal BC in this population is consistent with a recent case-control study reporting a positive association between high BMI and an increased risk of estrogen receptor and progesterone receptor positive BC (OR = 1.83; 95% CI: 1.08–3.09; *P* trend = 0.03) comparing the highest (>35 kg/m^2^) to the lowest (<25 kg/m^2^) quintile [30]. Other studies on women of African descent do not support a positive association between BMI and postmenopausal BC [34, 35, 40, 49]. In a prospective study among African Americans, Palmer et al. reported a nonstatistically significant decreasing trend for women with BMI of 35 kg/m^2^ or above compared to <25 kg/m^2^ [35]. Similarly, a recent case-control study including 505 postmenopausal BC cases and 278 controls found a RR of 0.76; CI (0.48–1.21) for women with BMI ≥ 28 kg/m^2^ compared to <21 kg/m^2^ [34]. Few studies on the association between obesity and BC have been conducted in Hispanics, of those, most have reported inconsistent association between BMI and risk of BC in postmenopausal women [50, 68, 69]. Recently, Sarkissyan et al. (2011) reported in a cross-sectional study, a lack of association between obesity measured by BMI and postmenopausal BC among Hispanic women in the US population (OR = 1.4, 95% CI: 0.5, 4.1) [51]. The heterogeneity among these findings may be due to the lack of power in African studies due to the small number of cases, in particular in postmenopausal women, reflecting the demographic structure of African populations. Another possible explanation for these differences may be the selection bias mainly in case-control studies, where anthropometric measurements are often obtained after diagnosis and may be affected by the effects of cancer development on the general condition of the patient. The lack of association between BMI and BC risk observed in Hispanic women may reflect different effects of fat accumulation and distribution in this Hispanic population compared to populations in high resource countries.

### 4.2. WHR and Breast Cancer Risk

**Figure 2:**
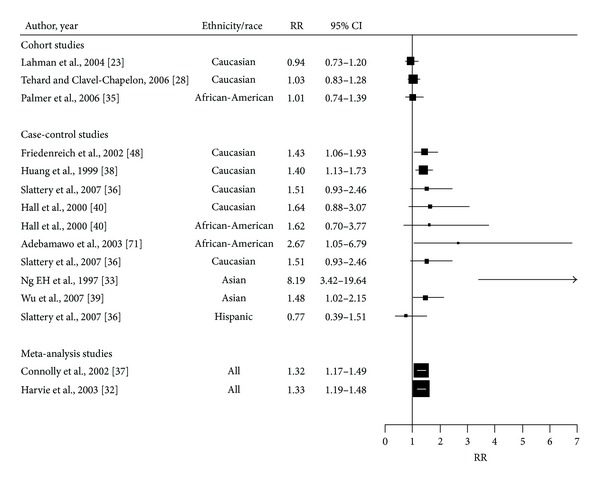
Forest plot of the association between WHR and breast cancer risk in postmenopausal women. The size of each box indicates the relative weight of each study; the horizontal bars show the 95% confidence intervals (CI). RR: relative risk; 95% CI: 95% confidence intervals.

WHR is commonly used as a measure of central obesity [31, 32, 70], defined as waist-hip ratio above 0.90 for males and above 0.85 for females has often been associated with the risk of developing postmenopausal BC (Table 2). However, while most studies have reported a significant increased risk [33, 37–39, 48], some studies are inconclusive [23, 35, 40] (Figure 2). A meta-analysis with six case-control and five cohort studies observed a summary risk estimate of 1.50 (95% CI: 1.10–2.04) for postmenopausal women [37]. The associations tend to be stronger in Asian women than other ethnic groups [33]. In a Canadian case-control study, a positive increase in RR of 1.43 (95% CI: 1.07–1.93) was observed when comparing the highest (>0.83) to the lowest quintile (<0.75) [48]. Similarly, in the Carolina case-control study, the OR for postmenopausal women in the highest quintile (>0.8) compared to lowest quintile (≤0.8) was 1.40 (95% CI: 1.1–1.7) [38]. A study in Africa (Nigeria) reported an RR of 2.67; 95 CI (1.05–6.80) for postmenopausal women with WHR > 0.85 compared to those with WHR < 0.77 [71]. In contrast, some studies conducted in the US did not detect a significant association. Hall et al. reported a non increased RR of 1.62; 95% CI (0.70–3.79) in African American women and of 1.64; 95% CI (0.88, 3.07) for Caucasian American women when comparing highest versus lowest quintiles (0.86–1.34 versus 0.6–0.77) [40]. However the power of the study was limited by the small number of cases (179 cases and 182 controls in African women). Regarding Hispanic women, only one study has assessed the association between WHR and BC risk. This study found no significant association between WHR and postmenopausal BC risk [36] (Figure 2).

## 5. In Premenopausal Women

### 5.1. BMI and Breast Cancer Risk

**Figure 3:**
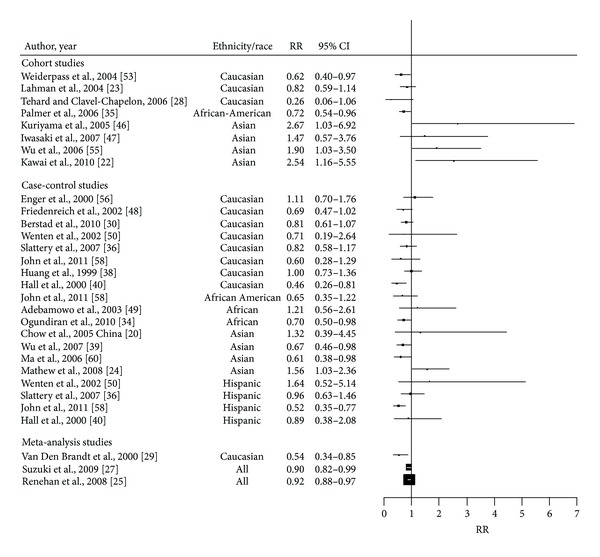
Forest plot of the association between BMI and breast cancer risk in premenopausal women. The size of each box indicates the relative weight of each study; the horizontal bars show the 95% confidence intervals (CI). RR: relative risk; 95% CI: 95% confidence intervals.

Previous studies, reviews, and pooled analyses have addressed the association of BMI and premenopausal BC (Table 3).  Figure 3 summarizes studies of the association between BMI and breast cancer risk in premenopausal women. In 2000, Van den Brandt et al. analyzed the association between premenopausal BC and BMI using pooled data from seven prospective cohort studies in Caucasian women. Multivariate analyses controlling for reproductive, dietary, and other risk factors detected that when compared to premenopausal women with a BMI of less than 21 kg/m^2^, women with a BMI exceeding 31 kg/m^2^ had an RR of 0.54 (95% CI: 0.34–0.85), suggesting an inverse association between BMI and risk of premenopausal BC [29]. A dose-response meta-analysis on BMI and premenopausal BC was conducted by Renehan et al. using a dataset of 20 studies. This analysis detected an overall risk estimate of 0.92 (95% CI: 0.88–0.97) for each increment of 5 kg/m² [25]. Several studies supported the hypothesis that higher level of BMI may be associated with a decrease in the risk of premenopausal BC. This hypothesis is supported by results from several case-control studies [34, 39, 40, 60] and cohort studies [35, 53]. However, others studies did not observe a statistically significant association when comparing highest versus lowest levels of BMI [23, 49, 56]. Ethnicity appears to modify the association of overweight, obesity, and BC. While the inverse association between BMI and risk of premenopausal BC is well documented in Caucasians, the association among Asian women is inconsistent. Several studies among Asian women suggest that higher BMI may be associated with an increased risk for premenopausal BC [22, 24, 46, 55]. A prospective study including 11,889 women from Taiwan reported that higher BMI was moderately associated with an increased risk of premenopausal BC [55], with an OR of 1.90 (1.00–3.4) for BMI > 26.2 kg/m^2^ versus 21.6 kg/m^2^. Similarly a multi-centric case-control study conducted among urban and rural women and including 898 cases and 1,182 controls reported an increased risk of 33–56% when comparing premenopausal women above 25 kg/m² with those with than less 25 kg/m² (OR = 1.33; 95% CI: 1.50–1.62 for BMI 25–29.9 kg/m² and 1.56; 95% CI: 1.03, 2.35 for BMI greater than 30 kg/m²) [24]. Interestingly one recent cohort study conducted in Japan reported a high increased risk of 2.54; 95% CI (1.16–5.55) when comparing women of BMI 25 kg/m² to those of <20 kg/m² [22]. In contrast, other studies among Asian women did not detect a significant association between BMI and the risk of premenopausal BC [20, 47]. Among African women, the majority of studies reported an inverse association between obesity and premenopausal BC. A case-control study conducted in Nigeria reported a significant decreasing trend in premenopausal BC with increasing BMI (*P* trend = 0.027) [34]. A similar finding was observed in a large prospective study conducted among African Americans in whom the multivariate adjusted RR was 0.72 (95% CI: 0.54–0.96) for BMI > 25 kg/m^2^ relative to BMI < 20 [35]. In contrast, some studies did not find a significant association [30, 40]. With respect to Hispanics, a case-control study conducted on Hispanic women living in the US concluded that high BMI at 30 years of age was associated with a decrease of the risk of premenopausal BC (OR = 0.46, 95% CI: 0.25, 0.84 for BMI > 30 versus <25 kg/m²) [36]. A more recent study has shown that high BMI was inversely associated with risk of BC in Hispanic population (*P* trend < 0.01) [58]. 

### 5.2. WHR and Breast Cancer Risk

**Figure 4:**
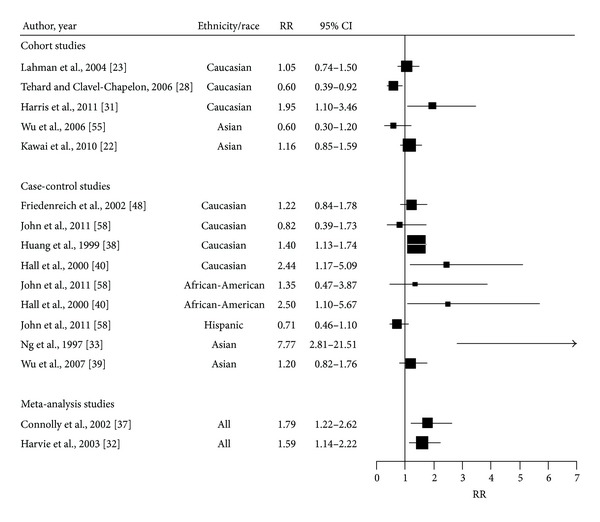
Forest plot of the association between WHR and breast cancer risk in premenopausal women. The size of each box indicates the relative weight of each study; the horizontal bars show the 95% confidence intervals (CI). RR: relative risk; 95% CI: 95% confidence intervals.

WHR has not been consistently associated with increased BC risk in premenopausal women, for whom both null [23, 38, 39] and increased risk have been reported [31, 32, 37, 40] (Figure 4, Table 3). Two meta-analyses [32, 37] have reported that a greater WHR was associated with about 1.5 fold increased risk of premenopausal BC. A pooled analysis on seven cohorts and four case-controls reported a summary risk estimate of 1.79; 95% CI (1.22–2.62) [37] but the strength of the association varied according to ethnic groups [41–43, 64]. A large prospective cohort study of 11,889 women conducted in Taiwan found that central adiposity reflected by hip circumference was a significant predictor of BC [55]. Similarly, in a prospective case-control study involving 1,086 Chinese women in Singapore, central obesity as indicated by a larger WHR was associated with highest risk for BC, with the OR being 7.81; (95% CI: 2.8–21.9) comparing the last (>0.86) and first quintile (<0.75), whereas BMI did not significantly predict the risk for BC [33]. Among Caucasian women, a case-control study including 523 cases and 471 controls, adjusted for BMI, reported an increase risk of 1.04 (1.01–1.08) for each increment of 0.1 unit of WHR and a risk of 2.44; 95% CI (1.17–5.09) when comparing the (0.86–1.34) versus (0.6–0.77) quintiles [40]. Few studies have been conducted on African women (African American and African); among those, a case-control study conducted among African American found a positive association between WHR and premenopausal BC (RR = 2.50; 95% CI: 1.1, 5.67, *P* trend < 0.005) for women with WHR (0.86–1.34) compared to those with WHR (0.6–0.77) [40]. Other studies [55, 58, 72] did not find a statically significant association. Overall, this increased risk associated with larger WHR among premenopausal women is found to be stronger amongst Asian women compared to other ethnic groups. Studies conducted between WHR and premenopausal BC among Hispanic women are limited and none has reported significant association. For example, John et al., reported a non significant decrease risk of having greater WHR among Hispanic women (OR = 0.71, 95% CI: 0.46–1.1; *P* trend = 0.19) [58]. 

## 6. Significance of Waist-to-Height Ratio

There is evidence suggesting that the waist-to-height ratio (WHtR) may be a more useful global clinical screening tool than WC, with a weighted mean boundary value of 0.5 [73]. The WHtR is defined as waist (cm) circumference divided by height (cm) and correlates well with abdominal obesity; higher values of WHtR indicate higher risk of obesity-related cardiovascular diseases [74]. However, to date the association between WHtR and BC risk has not been systematically studied. Nevertheless, a previous study of multiethnic groups reported an inverse association between WHtR and ER+PR+ BC in all premenopausal women [58].

## 7. Variations with Molecular Subtypes of Breast Cancer

The association between obesity and BC appears to vary according to the molecular subtype of BC, as defined by gene expression patterns into luminal A and B, HER2+, and triple-negative subtypes. A meta-analysis suggests that the association between BMI and BC risk is heterogeneous according to estrogen receptor (ER) and progesterone receptor (PR) status of the tumor. Higher BMI was associated with higher risk for ER+/PR+ tumors in postmenopausal women (RR = 1.82; 95% CI: 1.55–2.14) but had a protective effect on ER+/PR+ tumors in premenopausal women (RR = 0.80; 95% CI: 0.70–0.92). By contrast, no associations were observed for ER−PR− tumors [27]. In 2011, Harris et al. investigated the association between body fat distribution, assessed in 1993 by self-reported WC, HC, and WHR, and the incidence of premenopausal BC in the Nurses' Health Study II. Each of these body fat distribution measures appeared to be statistically significantly associated with greater incidence of estrogen receptor (ER) negative BC (RR for ER-negative BC for the highest versus the lowest quintile of WHR was 1.95 (95% CI: 1.10–3.46; *P* trend = 0.01) [31]. These findings suggest that body fat distribution may be associated with an increased risk for ER-negative BC among premenopausal women. 

In a pooled analysis of tumor marker and epidemiological risk factor data from 35,568 invasive BC case patients from 34 studies participating in the BC Association Consortium, obesity (BMI ≥ 30 kg/m^2^) in women ≤50 years was found to be more frequent in ER−/PR− than in ER+/PR+ tumors (*P* = 1.10^−7^). In contrast, obesity in women >50 years was less frequent in PR− than in PR+ tumors (*P* = 6.10^−4^) [75]. This study demonstrated that elevated BMI in younger women (≤50 years) was associated with the risk of ER+ or PR+ BC but not with ER−/PR− BC and suggested that triple-negative or core basal phenotype (CBP); defined by triple-negative and cytokeratins [CKs] 5/6+ (CBP) BC may have distinct etiology [75]. The differences between the conclusions of the two studies [31] may be linked to the use of different obesity/fat distribution measures. Further studies are needed to better understand the associations between overweight and obesity and specific subtypes of BC. 

## 8. Effect of Early Life Anthropometry on Breast Cancer Risk

Most of the epidemiological studies have focused mainly on adult BMI and not on weight change or on the influence of early life body weight and body shape (silhouette). These factors, such as birth size, body shape, and weight during childhood or adolescence may play a role in the risk of BC. Epidemiologic studies have demonstrated that independently of BMI, greater body fatness during childhood or adolescence are associated with lower BC risk in both premenopausal women [30, 36, 45, 53, 54, 57] and postmenopausal women [30, 35, 44, 45]. In a recent large prospective study conducted among 188,860 women, Baer and colleagues reported a strong protective effect of body fatness on childhood and adolescence among both premenopausal (RR = 0.91; 95% CI: 0.87–0.94 and 0.88; 95% CI: 0.87–0.94, *P* trend < 0.0001 per each 1-unit increase resp.) and postmenopausal BC (RR = 0.93; 95% CI: 0.90–0.95 and 0.91; 95% CI: 0.89–0.93), *P* trend < 0.0001 per each 1-unit increase resp.) [45]. In the same study, this inverse association was stronger for women with birth weights under 8.5 pounds (<3.9 kg) than for women with birth weights of 8.5 pounds or more (3.9 kg) [45]. These results are consistent with a recent study conducted among premenopausal Hispanic women in the US, which reported a significant inverse association with the relative weight compared to peers, at age 10 years (OR = 0.63 (95% CI: 0.33–1.20, *P* trend = 0.005), at age 15 years (OR = 0.31 (95% CI: 0.16–0.6, *P* trend < 0.001), and at age 20 years (OR = 0.44 (95% CI: 0.24–0.84, *P* trend = 0.002) when compared heavier versus lighter weight [59]. Interestingly, in the same Hispanic study, when considering the joint effects of current BMI and adolescent body size, among overweight women (BMI ≥ 25 kg/m^2^), the risk of premenopausal BC decreased with heavier relative weight at ages 15 years (OR = 0.26; 95% CI: 0.12–0.56, *P* trend < 0.01), 20 years (OR = 0.42; 95% CI: 0.21–0.87, *P* trend = 0.02) and heavy body shape (silhouette) at age 20 years (OR = 0.37; 95% CI: 0.17–0.79, *P* trend = 0.02). Increases in body fatness between childhood, adolescence, and age at diagnosis were also inversely associated with risk of premenopausal BC risk. The RRs were 0.77; 95% CI (0.61–0.98) for women who showed increased body fatness (as assessed by changes in body shape) from age 5 to 10 years and 0.82; 95% CI (0.67–0.99) from age 10 to 20 years, respectively [59]. These results suggest that the association between risk of BC and changes in body fatness or obesity in adolescence and childhood may be a stronger determinant of BC than recent weight. The biological mechanisms underlying the effects of changes in body fatness in adolescence and childhood on the risk of BC are still poorly understood. 

## 9. Metabolic Pathways for Breast Cancer Development

**Figure 5:**
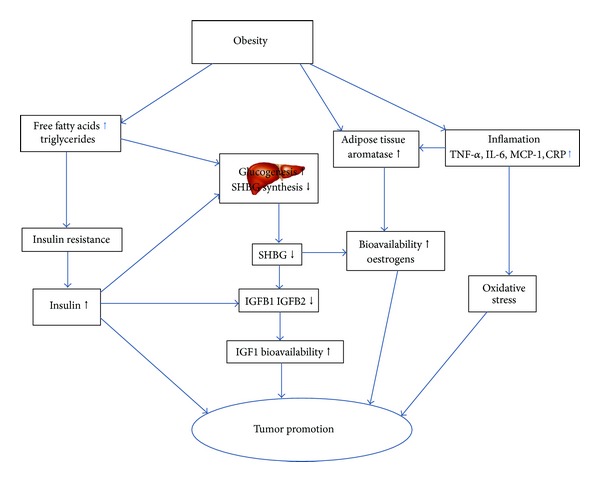
Metabolic pathways linking obesity and breast cancer risk.

The complex associations between anthropometric measures of body fatness/obesity and the risk of BC suggest that metabolic conditions associated with high body fatness may influence this risk in several ways, with distinct effects on pre- and postmenopausal BC, as well as on different molecular subtypes of BC. It is now commonly accepted that the occurrence and development of BC is driven by the abnormal, clonal expansion of pools of initiated stem/progenitor cells [76, 77]. The existence of different types of such progenitors, as well as the effect of molecular alterations allowing the progeny of these cells to switch from one differentiation phenotype to another, may account for a large part of the molecular and epidemiological heterogeneity of BC. According to this view, the risk of BC may depend upon whether specific pools of progenitor cells are either enhanced or suppressed at particular stages of breast maturation and development. Metabolic parameters associated with body fatness may affect these progenitor cells through five main biological mechanisms (Figure 5). First, they may affect the cell's bioenergetic balance and favor the expansion of cells with high anaerobic glycolytic capacity, a bioenergetics adaptation which characterizes cancer cells. This effect, known as “Warburg effect,” is defined by intense lipogenesis and glycolysis and low mitochondrial oxidative phosphorylation capability even in the presence of sufficient oxygen [78, 79]. Hyperinsulinemia and high blood glucose levels, which are frequent in obese subjects, are expected to provide a selective advantage for the growth of such cells. Second, increased adiposity may have an impact on sterol synthesis and on the metabolism of estrogens. Obesity (high BMI) has been associated with increasing sex hormone (estrogen) due to increased peripheral aromatization of adrenal androgens in adipose tissue among postmenopausal women, which can promote cell proliferation, anti-apoptotic and proangiogenic effects [80, 81]. Third, high blood levels of insulin and insulin-like growth factor (IGF-I) have been found to stimulate the growth and survival of cancer cells in both pre- and post-menopausal women and their production can be increased by estrogen [82–84]. Fourth, obesity induces chronic low-grade inflammation resulting in an increase of local and systemic levels of cytokines (such as TNF-*α*, interleukin-6 (IL-6), C-reactive protein (CRP), and monocyte chemoattractant protein-1 (MCP-1)). Obesity can also increase adipokines (leptin and adiponectin). These factors may, in turn, affect mitosis, apoptosis, angiogenesis, and cell migration and escape from immune recognition [85]. Fifth, increase in free fatty acids, such as triglycerides, has been reported to increase the level of free estradiol by displacing estradiol from sex hormone binding globulin (SHBG) [86]. Therefore, both decreases in SHBG and increases in triglycerides may result in increased free estradiol. 

The above mechanisms are not mutually exclusive. They may operate in a complementary manner to promote specific forms of BC. One of the main paradox is the apparently opposite association of body fatness with pre- and postmenopausal BC. In Caucasians, most postmenopausal BC are ER+/PR+ (luminal A) subtypes and the effect of body fatness may involve increased hormone biosynthesis in adipose after the menopause, leading to the long-term maintenance of breast progenitor cells after the menopause. In contrast, in pre-menopausal women, the apparently protective effect of obesity may be due to hormone-independent forms of BC, which are more common among premenopausal women. Also, in premenopausal women estradiol levels are reduced in anovulatory cycles that are more frequent in obese than lean women. In addition, obese premenopausal women have been found to have reduced progesterone levels [87], accounting for the negative association between BMI and risk of premenopausal BC. The lack of the association between estradiol levels and risk of BC development in premenopausal women suggest that obesity may affect breast progenitor cells through mechanisms other than estradiol levels.

## 10. Conclusion

Obesity has become a crucial public health problem worldwide, especially for BC development and survival. Most studies have shown that BMI which reflects general obesity is associated with a decrease of the risk of developing BC before menopause and increase after menopause in most of the studies, while WHR which reflects central obesity is associated with an increased risk of both pre- and postmenopausal BC. Results are consistent with differences in metabolic risk and definitions of obesity according to ethnicity. Data regarding the relationship between obesity and young age and BC have demonstrated a strong inverse association between body fatness during childhood and adolescent and risk of BC throughout life in Caucasian population. The mechanisms for this inverse association are not fully understood and need further research. It will also be important to develop stringent recommendations and to maintain a healthy weight both at individual and community levels.

## Figures and Tables

**Table:** (a)

	Body mass index	Obesity class	Disease risk (relative to normal weight and waist circumference)	
			Men < 102 cm	Men > 102 cm
			Women < 88 cm	Women > 88 cm

Underweight	<18.5			
Normal	18.5–24.9			
Overweight	25.0–29.9		Increased	High
Obesity	30.0–34.9	I	High	Very high
	35.0–39.9	II	Very high	Very high
Extreme obesity	>40	III	Extremely high	Extremely high

**Table:** (b)

Indicator	Cut-off points	Risk of metabolic complications
Waist circumference	Men > 94 cm; women > 80 cm	Increased
Waist circumference	Men > 102 cm; women > 88 cm	Substantially increased
Waist-hip ratio	Men ≥ 0.90 cm; women ≥ 0.85 cm	Substantially increased

**Table 2:** Selected characteristics of studies in postmenopausal women included in the paper.

First author, year, and country	Study design	Population	Cases/controls or P-years	Type of exposure	Range of exposure	RR (95% CI)	Variables of adjustment or comment
Lahmann 2004 Germany [23]				BMI			Study center, age, educational attainment, smoking status, alcohol consumption, parity, age at first pregnancy, age at menarche, and current pill use
				non-HRT	>30 versus <25	**1.31 **(**1.08–1.59**)	
	Cohort	Caucasian	1,405/103,344	HRT	>30 versus <25	**0.66 **(**0.45–0.98**)	
				WHR			
				non-HRT	>0.84 versus <0.73	0.94 (0.74–1.21)	
				HRT	>0.84 versus <0.73	0.85 (0.60–1.20)	
Tehard 2006 France [28]	Cohort	Caucasian	860/41497	BMI WHR	≥30 versus <18.5 ≥0.82 versus 0.74	**1.44 **(**1.04–1.99**) 1.03 (0.83–1.28)	FHBC, age at menarche, age at first birth, parity, history of benign breast disease, alcohol consumption, number of years of education, marital status, and physical activity
Bardia 2008 United States [44]	Cohort	Caucasian	2503/35941	Weight at age 12	Above versus average	**0.85 **(**0.74–0.98**)	Age, education status, age at menopause, age at menarche, parity, age at first birth, BMI at age 18 years, OC, HRT, smoking, alcohol, and physical activity level
Baer 2010 United States [45]	Cohort	Caucasian	4974/188,860	Body fatness Childhood 5–10 Y Adolescent 10–20 Y	0.1 unit increase 0.1 unit increase	**0.93 **(**0.90–0.95**) **0.91 **(**0.89–0.93**)	Age, time period, parity/age at first birth, FHBC, personal history of benign BC, height, alcohol intake, OC use, birth weight, age at menopause, and HRT use
Kuriyama 2005 Japan [46]	Cohort	Asian	65/9,666	BMI	≥30 versus <25	**2.67 **(**1.03–6.92**)	Age, smoking, alcohol, consumption of meat, fish, fruits, green or yellow consumption of bean-paste soup, type of health, parity, age at menarche, and age at FFT pregnancy
Iwasaki 2007 Japan [47]	Cohort	Asian	441/55,537	BMI	>30 versus <19	2.28 (0.94–5.53)	Age, area, number of births, age at first birth, and height
Kawai 2010 Japan [22]	Cohort	Asian	108/10,106	BMI	≥25 versus <20	**2.54 (1.16–5.55)**	Age, education, smoking, alcohol, and time spent walking, Menstrual and reproductive factors, HRT, and FHBC
Palmer 2007 United States [35]	Cohort	African-American	455/59,000	BMI at age 18 Current BMI WHR	≥25 versus <20 ≥37 versus <25 ≥0.87 versus <0.71	**0.55 (0.37–0.82**) 0.78 (0.58–1.05) 1.01 (0.74–1.40)	Age, age at menarche, parity, age at first birth, and family history of breast cancer
Friedenreich 2002 Canada [48]	Case-control	Caucasian	771/762	BMI WHR	≥31.3 versus <24.1 ≥0.83 versus <0.75	0.99 (0.74–1.32) **1.43 **(**1.07–1.93**)	Age, total caloric intake, physical activity, educational level, HRT, diagnosed with benign BC, FHBC, alcohol, and current smoke
Huang 1999 United States [38]	Case-control	Caucasian African-American	436/354	WHR	>0.8 versus ≤0.8	**1.40 **(**1.10–1.70)**	Age at menarche, nulliparity, breastfeeding, Abortion or miscarriage, BMI, WHR, oral contraceptive, HRT, FHBC, smoking, alcohol, education, medical radiation to the chest
				BMI			
				Black	30.1–59.2 versus 14.6–24.6	0.68 (0.33–1.42)	
Hall 2000 United States [40]	Case-control	Caucasian African-American	382/419	White	30.1–59.2 versus 14.6–24.6	1.08 (0.58–2.00)	Age, age at menarche, parity/age at FFT pregnancy, lactation, and education
				WHR			
				Black	0.86–1.34 versus 0.6–0.77	1.62 (0.70–3.79)	
				White	0.86–1.34 versus 0.6–0.77	1.64 (0.88–3.07)	
				BMI at 18 years	≥25 versus <20	**0.72 (0.55–0.96)**	
Berstad 2010 United States [30]	Case-control	Caucasian African-American	1,900/2,006	Recent BMI			Age, race, education, study site, FHBC, parity, age at menopause, and HRT
				Caucasian	≥35 versus <25	0.75 (0.53–1.06)	
				African-American	≥35 versus <25	1.26 (0.55–1.85)	
Ogundiran 2010 Nigeria [34]	Case-control	African	498/266	BMI	≥28 versus <21	0.76 (0.48–1.21)	Age, ethnicity, education, menarche, parity, age at FFTP, breastfeeding, menopausal status, age at menopause, FHBC, benign breast disease, contraceptive, alcohol
Adebamowo 2003 Nigeria [49]	Case-control	African	234/273	BMI	≥30 versus <20	1.82 (0.78–4.31)	Age, age at menarche, age at FFT pregnancy, height
Wenten 2002 United States [50]	Case-control	Caucasian Hispanic	687/820	BMI			Age; FHBC; total MET-hours; parity; OC; breastfeeding; age at first live birth; months of HRT use
				Caucasian	≥30 versus <22	2.77 (0.86–8.89)	
				Hispanic	≥30 versus <22	1.32 (0.47–3.72)	
				BMI non-HRT user			Age, center, physical activity level, energy intake, alcohol intake, age at menopause, parity, and Height
				Caucasian	>30 versus <25	**1.61** (**1.05–2.45**)	
Slattery 2007 United States [36]	Case-control	Caucasian Hispanic	2,325/2,525	Hispanic	>30 versus <25	0.80 (0.44–1.45)	
				WHR non-HRT user			
				Caucasian	>0.9 versus <0.8	1.51 (0.93–2.46)	
				Hispanic	>0.9 versus <0.8	0.77 (0.39–1.50)	
Ng 1997 Singapore [33]	Case-control	Asian	130/585	WHR	>0.86 versus <0.75	**8.19 (3.40–19.50)**	Age, menopausal status, age at menarche, parity, number of birth, age at FFT birth, HRT, OC, breast feeding, smoking, height, weight, and BMI
Chow 2005 China [20]	Case-control	Asian	198/353	BMI	23–27 versus <19 <31 versus <19	**1.73 (1.04–2.86)** **3.82 (1.03–14.27)**	Age, number of pregnancies, family history of breast cancer, income, smoking, alcohol, use of OC, and education
Wu 2007 Japan [39]	Case-control	Asian	1,277/1,160	BMI WHR	>24.60 versus ≤20.43 >0.84 versus ≤0.76	1.35 (0.95–1.93) **1.48 (1.02–2.15)**	Age, Asian ethnicity, duration of residence in the US, education, menarche, parity, menopausal status, age at menopause, intake of tea and soy, and physical activity
Mathew 2008 India [24]	Case-control	Asian	968/691	BMI	25–29.9 versus <25 >30 versus <25	**1.29 (1.00–1.66)** 1.00 (0.64–1.54)	Age, center, religion, marital status, education socioeconomic status, residence status, parity, age at 1st childbirth, duration of breast feeding, and physical activity
Connolly 2002 Canada [37]	Meta-analysis	All	19 studies	WHR	0.1 unit increase	**1.50 (1.10–2.04) **	A meta-analysis was done to summarize the literature on WHR and breast cancer risk published from January 1966 to August 2002
Harvie 2003 United kingdom [32]	Meta-analysis	All	8 studies: 5 cohort and 3 case-control	WHR			Lifestyles and reproductive factors (confounders that were found to be significant in proportional hazard regression analysis)
				Cohort studies	0.75 versus 0.80	**1.32** (**1.16–1.49**)	
				Case-control studies	0.75 versus 0.80	1.82 (0.85–3.85)	
Suzuki 2009 Sweden [27]	Meta-analysis	All	31 studies: 9 cohort and 22 case-control	BMI ER+PR+	0.5 unit increase	**1.33 (120–1.48)**	Meta-analysis of cohort and case-control studies (from 1970 to 2007) was performed to clarify the association between body weight and the incidence of BC defined by ER/PR status of the tumors
Van Den Brandt 2000 United States [29]	Meta-analysis	Caucasian	4,385/337,819	BMI	21 versus 31	**1.26 (1.09–1.46)**	HRT, OC, history of benign breast disease, FHBC, smoking status, education, fat intake, fiber intake, energy intake, and alcohol intake
Renehan 2008 United States [25]	Meta-analysis	Caucasian Asian	31 studies	BMI all North American European and Australian Asia-Pacific	5 units increase 5 units increase 5 units increase 5 units increase	**1.12 (1.08–1.16)** **1.15 (1.08–1.23)** **1.09 (1.04–1.48)** **1.31 (1.15–1.48)**	Cohort and case-control studies published from 1966 to November 2007 were included in the analysis. The dose response meta-analysis was adjusted by geographic region and cancer site
Sarkissyan 2011 United States [51]	Cross- sectional	African-American Hispanic	237/234	BMI			Age, ethnicity, comorbidity, and menopausal status
				African-American	≥30 versus <25	4.8 (1.8–12.7)	
				Hispanic	≥30 versus <25	1.4 (0.5–4.1)	

BMI: measurement of body mass index (in kg/m^2^); WHR: waist-hip ratio; ER: estrogen receptor; PR: progesterone receptor; BC: breast cancer; HRT: hormonal replacement therapy; OC: oral contraceptives; FFT: age at first full-term pregnancy; FHBC: family history of breast cancer; Y: years.

Bold: statistically significant.

**Table 3:** Selected characteristics of studies in premenopausal women included in the paper.

Authors, years, and country	Study design	Population	Cases/controls or P-years	Type of exposure	Range of exposure	RR (95% CI)	Variables of adjustment or comments
Weiderpass et al., 2004 Norway-Sweden [53]	Cohort	Caucasian	733/99,717	BMI BMI at age 18 Body shape at age 7	**>30 versus <20** **≥25 versus 20–24.9** **Fat/very fat versus average**	**0.62 (0.40–0.97)** **0.74 (0.59–0.91)** **0.69 (0.50–0.93)**	Age at enrolment, parity, age at FFT pregnancy, OC, age at menarche, FHBC, total duration of breast feeding, and country
Lahmann et al., 2004 Germany [23]	Cohort	Caucasian	474/73,542	BMI WHR	≥28.8 versus <21.5 >0.846 versus <0.736	0.82 (0.59–1.14) 1.05 (0.74–1.50)	Study center, age, educational attainment, smoking, alcohol, parity, age at first pregnancy, age at menarche, and current pill use
Baer et al., 2005 United States [54]	Cohort	Caucasian	1,318/109,267	Increase body fatness			Age, time period, birth weight, height, recent alcohol consumption, parity, age at first birth, OC, history of benign breast disease, and first degree of FHBC
				From age 5 to 10	≥2 levels versus no change	**0.77 (0.61–0.98)**	
				From age 5 to 20	≥2 levels versus no change	**0.77 (0.65–0.91)**	
				From age 10 to 20	≥2 levels versus no change	**0.82 (0.67–0.99)**	
Tehard and Clavel-Chapelon, 2006 France [28]	Cohort	Caucasian	275/20,068	BMI WHR	>30 versus <18.5 ≥0.82 versus 0.74	**0.26 (0.06–1.00)** **0.60 (0.39–0.91) **	FHBC, age at menarche, age at FFTP, parity, history of benign breast disease, alcohol consumption, education, marital status, and physical activity
Baer et al., 2010 United States [45]	Cohort	Caucasian	2,188/188,860	Body fatness Childhood 5–10 Y Adolescent 10–20 Y	0.2 unit increase 0.1 unit increase	0.91 (0.87–0.94) 0.88 (0.87–0.94)	Age, time period, parity, age at first birth, FHBC, personal history of benign breast disease, height, alcohol intake, OC, and birth weight
Harris et al., 2011 United States [31]	Cohort	Caucasian	620/45,799	WHR ER−	≥0.84 versus <0.73	**1.95 (1.10–3.46)**	Age, height, history of benign breast disease, FHBC, age at menarche, age at FFTP, parity, OC, alcohol, and physical activity
Kuriyama et al., 2005 Japan [46]	Cohort	Asian	33/5,214	BMI	≥29.9 versus <25 ≥30 versus <25	0.84 (0.24–2.88) **2.67 (1.03–6.92)**	Age, smoking, alcohol, consumption of meat, fish, fruits, green or yellow consumption of bean-paste soup, type of health, parity, age at menarche, and age at FFT pregnancy
Iwasaki et al., 2007Japan [47]	Cohort	Asian	441/55,537	BMI	>30 versus <19	1.47 (0.53–3.47)	Age, area, number of births, age at first birth, and height
Wu et al., 2006 Taiwan [55]	Cohort	Asian	104/11,889	BMI WHR	>26.2 versus 21.6 <0.85 versus <0.77	1.90 (1.00–3.40) 0.60 (0.30–1.20)	Age at enrollment, height, weight
Kawai et al., 2010 Japan [22]	Cohort	Asian	108/10,106	BMI	≥25 versus <20	**2.54 (1.16–5.55)**	Age, education level, smoking, alcohol, time spent walking, which are known or suspected risk factors for BC. Menstrual and reproductive factors, HRT, and FHBC
Palmer et al., 2007 United States [35]	Cohort	African-American	496/59,000	BMI at age 18 years Current BMI WHR	≥25 versus <20 ≥37 versus <25 ≥0.87 versus <0.71	**0.68 (0.46–0.87)** **0.72 (0.54–0.96)** 1.16 (0.85–1.59)	Age, age at menarche, parity, age at first birth, FHBC
Enger et al., 2000 United States [56]	Case-control	Caucasian	701/714	BMI			Age at reference year, socioeconomic status, age at menarche, age at FFT, number of full-term pregnancies, months of breastfeeding
				ER+PR+	>32.4 versus <17.36	1.11 (0.7–1.77)	
				ER+PR−	>32.4 versus <17.36	0.92 (0.34–2.47)	
				ER−PR−	>32.4 versus <17.36	1.07 (0.56–1.68)	
Friedenreich et al., 2002 Canada [48]	Case-control	Caucasian	462/475	BMI WHR	≥29.2 versus <23.1 ≥0.81 versus <0.72	0.69 (0.47–1.02) 1.22 (0.84–1.79)	Age, total caloric intake, physical activity, educational level, HRT, diagnosed with benign BC, FHBC, alcohol, and smoking
Magnusson and Roddam, 2005 United kingdom [57]	Case-control	Caucasian	1560/1548	Body fatness at 10 Y Change between 10 and diagnosis	Plump versus thin Plump/overweight versus thin/thin	**0.83 (0.69–0.99)** **0.75 (0.56–1.01)**	Age and recruitment, region, parity, age at first birth, height, OC, and alcohol
Berstad et al., 2010 United States [30]	Case-control	Caucasian	2,097/2,035	BMI at 18 years Recent BMI	≥25 versus <20 ≥35 versus <25	**0.76 (0.63–0.90)** 0.81 (0.61–1.06)	Age, race, education, study site, family history, parity, age at menopause, and HT use
Wenten et al., 2002 United States [50]	Case-control	Caucasian Hispanic	687/820	BMI			Age; FHBC; total MET-hours; parity; OC; breastfeeding; and age at first live birth
				Caucasian	≥30 versus <22	0.71 (0.19–2.63)	
				Hispanic	≥30 versus <22	1.64 (0.52–5.11)	
Slattery et al., 2007 United States [36]	Case-control	Caucasian Hispanic	2,325/2,525	Current BMI			Age, height, physical activity, energy intake, parity, alcohol consumption, age at first pregnancy, and center
				Caucasian	>30 versus <25	0.82 (0.58–1.17)	
				Hispanic	>30 versus <25	0.96 (0.63–1.46)	
				BMI at 30 years			
				Caucasian	>30 versus <25	0.91 (0.52–1.60)	
				Hispanic	>30 versus <25	**0.46** (**0.25–0.84**)	
John et al., 2011 United States [58]	Case-control	Caucasian African-American Hispanic	672/808	BMI all African-American Caucasian Hispanic WHR African-American Caucasian Hispanic	>30 versus <25 >30 versus <25 >30 versus <25 >30 versus <25 >0.85 versus ≤0.77 >0.85 versus ≤0.77 >0.85 versus ≤0.77 >0.85 versus ≤0.77	**0.60** (**0.45–0.79**) 0.65 (0.35–1.23) 0.60 (0.28–1.30) **0.52** (**0.35–0.77**) 0.78 (0.56–1.08) 0.82 (0.39–1.74) 1.35 (0.47–3.86) 0.71 (0.46–1.11)	Age, country of birth, education level, FHBC biopsy-confirmed history of benign breast disease, age at menarche, parity, breastfeeding, alcohol, physical activity, daily caloric intake, and height. Analyses of all women combined were also adjusted for race/ethnicity
Ng et al., 1997 Singapore [33]	Case-control	Asian	74/297	WHR	>0.86 versus <0.75	**7.81 (2.8–21.9)**	Age, menopausal status, age at menarche, parity, number of birth, age FFT birth, HR, OC, breast feeding, smoking, height, weight, BMI
Chow et al., 2005 China [20]	Case-control	Asian	198/353	BMI	27–31 versus <19	1.32 (0.39–4.43)	Age, number of pregnancies, FHBC, income, smoking, alcohol, use of OC, education
Sangaramoorthy et al., 2011 United States [59]	Case-control	Hispanic	210/265	Weight at age 10 Weight at age 15 Weight at age 20	Heavier versus lighter Heavier versus lighter Heavier versus lighter	0.63 (0.33–1.20) **0.31 (0.16–0.61)** **0.44 (0.24–0.84)**	Age, country of birth, education, FHBC, prior biopsy-confirmed history of benign breast disease, parity, lifetime, breastfeeding, age at FFT, OC, adult height, alcohol consumption, and average caloric intake
Huang et al., 1999 United States [38]	Case-control	Caucasian African-American	436/354	BMI all ER+PR+ ER−PR− WHR all ER+PR+ ER−PR−	>31 versus <31 >31 versus <31 >31 versus <31 >0.8 versus ≤0.8 >0.8 versus ≤0.8 >0.8 versus ≤0.8	1.00 (0.70–1.30) 1.10 (0.70–1.70) 0.70 (0.40–1.20) **1.40 (1.10–1.70)** **1.40 (1.00–1.90)** **1.40 (0.90–2.00)**	Age at menarche, nulliparity, breastfeeding, abortion or miscarriage, BMI, WHR, oral contraceptive, HRT, FHBC, smoking, alcohol, education, medical radiation to the chest
Hall et al., 2000 United States [40]	Case-control	Caucasian African-American	390/319	BMI			Age, age at menarche, parity/age at FFT pregnancy, lactation, education
				Black	14.6–24.6 versus 30.1–58.2	0.89 (0.38–2.07)	
				White	14.6–24.6 versus 30.1–58.2	**0.46 (0.26–0.80)**	
				WHR			
				Black	0.6–0.77 versus 0.86–1.34	**2.50 (1.10–5.67)**	
				White	0.6–0.77 versus 0.86–1.34	**2.44 (1.17–5.09)**	
Adebamowo et al., 2003 Nigeria [49]	Case-control	African	234/273	BMI	≥30 versus <20	1.21 (0.56–2.60)	Age, age at menarche, age at first pregnancy, height
Ogundiran et al., 2010 Nigeria [34]	Case-control	African	707/820	BMI	31.2 versus <19.5 ≥28 versus <21	**0.70 (0.50–0.98)** 0.76 (0.48–1.21)	Age, ethnicity, education, age at menarche, number of live births, age at first live birth, duration of breastfeeding, menopausal status, FHBC, benign BC, OC, and alcohol
Wu et al., 2007 Asian [39]	Case-control	Asian	1,277/1,160	BMI WHR	>24.60 versus ≤20.43 >0.84 versus ≤0.76	**0.67 (0.46–0.98)** 1.20 (0.82–1.77)	Age, Asian ethnicity, duration of residence in the US, education, age at menarche, number of live births, menopausal status, intake of tea and soy during adolescence and adult life, and physical activity
Ma et al., 2006 United States [60]	Case-control	Asian	1,725/440	BMI	≥35 versus <25	**0.61 (0.38–0.99)**	Race, age, education, first-degree FHBC, age at menarche, gravity, number of full-term pregnancy, BMI 1 year before reference date, OC
Mathew et al., 2008 India [24]	Case-control	Asian	898/1,182	BMI	25–29.9 versus <25 >30 versus <25	**1.33 (1.50–1.62)** **1.56 (1.03–2.35**)	Age, center, religion, marital status, education, socioeconomic status, residence status, parity, age at 1st childbirth, breast feeding, and physical activity
Connolly et al., 2002 Canada [37]	Meta-analysis	All	19 studies	WHR	0.1 unit increase	**1.79 (1.22–2.62)**	A meta-analysis was done to summarize literature on WHR and breast cancer risk published from January 1966 to August 2002
Harvie et al., 2003 United kingdom [32]		All	8 studies: 5 cohort and 3 case-control	WHR			Lifestyles and reproductive factors (confounders that were found to be significant in proportional hazard regression analysis)
	Meta-analysis			Cohort studies	>0.80 versus <0.75	**1.59** (**1.14–2.22**)	
				Case-control studies	>0.80 versus <0.75	**2.70** (**1.52–4.76**)	
Suzuki et al., 2009 Sweden [27]	Meta-analysis	All	31 studies: 9 cohort and 22 case-control	BMI ER+PR+	5 units increase	0.90 (0.82–0.99)	Meta-analysis of cohort and case-control studies (from 1970 to 2007) between body weight, and the incidence of BC defined by ER/PR status
Van Den Brandt et al., 2000 United States [29]	Meta-analysis	Caucasian	7 cohort studies	BMI	>31 versus <21	**0.54 (0.34–0.85)**	OC use, history of benign BC, FHBC, smoking status, education, fat intake, fiber intake, energy intake, and alcohol intake
Renehan et al., 2008 United States [25]	Meta-analysis	Caucasian Asian	20 studies 79,30/2, 559,829	BMI (all)	5 units increase	**0.92 (0.88–0.97)**	Cohort and case-control studies published from 1966 to November 2007 were included in the analysis. The dose response meta-analysis was adjusted by geographic region and cancer site
				North American	5 units increase	**0.91 (0.85–0.98)**	
				European and Australian	5 units increase	**0.89 (0.84–0.94**)	
				Asia-Pacific	5 units increase	**1.16 (1.01–1.32)**	

BMI: measurement of body mass index (in kg/m^2^); WHR: waist-hip ratio; ER: Estrogen receptor; PR: progesterone receptor; BC: breast cancer; HRT: hormonal replacement therapy; OC: oral contraceptives; FFT: age at first full term pregnancy; FHBC: family history of breast cancer; Y: years.

Bold: statistically significant.

## Abbreviations

BMI: Body mass index
BC: Breast cancer
CI: Confidence interval
ER−: Estrogen negative
HER2, HC: Hip circumference
FHBC: Family history of breast cancer
IGF-1: Insulin-like growth factor 1
OR: Odd ratio
SES: Socioeconomic status
US: United States
WC: Waist circumference
WHR: Waist hip ratio
WHO: World Health Organization.
